# The influence of anthropogenic habitat fragmentation on the genetic structure and diversity of the malaria vector *Anopheles cruzii* (Diptera: Culicidae)

**DOI:** 10.1038/s41598-020-74152-3

**Published:** 2020-10-22

**Authors:** Laura Cristina Multini, Ana Letícia da Silva de Souza, Mauro Toledo Marrelli, André Barretto Bruno Wilke

**Affiliations:** 1 Department of Epidemiology, School of Public Health, University of São Paulo, São Paulo, SP Brazil; 2 São Paulo Institute of Tropical Medicine, University of São Paulo, São Paulo, SP Brazil; 3 Department of Public Health Sciences, Miller School of Medicine, University of Miami, 1120 Northwest 14th Street, Miami, FL 33136 USA

**Keywords:** Computational biology and bioinformatics, Evolution, Genetics, Molecular biology

## Abstract

Fragmentation of natural environments as a result of human interference has been associated with a decrease in species richness and increase in abundance of a few species that have adapted to these environments. The Brazilian Atlantic Forest, which has been undergoing an intense process of fragmentation and deforestation caused by human-made changes to the environment, is an important hotspot for malaria transmission. The main vector of simian and human malaria in this biome is the mosquito *Anopheles cruzii*. Anthropogenic processes reduce the availability of natural resources at the tree canopies, *An. cruzii* primary habitat. As a consequence, *An. cruzii* moves to the border of the Atlantic Forest nearing urban areas seeking resources, increasing their contact with humans in the process. We hypothesized that different levels of anthropogenic changes to the environment can be an important factor in driving the genetic structure and diversity in *An. cruzii* populations. Five different hypotheses using a cross-sectional and a longitudinal design were tested to assess genetic structure in sympatric *An. cruzii* populations and microevolutionary processes driving these populations. Single nucleotide polymorphisms were used to assess microgeographic genetic structure in *An. cruzii* populations in a low-endemicity area in the city of São Paulo, Brazil. Our results show an overall weak genetic structure among the populations, indicating a high gene flow system. However, our results also pointed to the presence of significant genetic structure between sympatric *An. cruzii* populations collected at ground and tree-canopy habitats in the urban environment and higher genetic variation in the ground-level population. This indicates that anthropogenic modifications leading to habitat fragmentation and a higher genetic diversity and structure in ground-level populations could be driving the behavior of *An. cruzii,* ultimately increasing its contact with humans. Understanding how anthropogenic changes in natural areas affect *An. cruzii* is essential for the development of more effective mosquito control strategies and, on a broader scale, for malaria-elimination efforts in the Brazilian Atlantic Forest.

## Introduction

Anthropogenic interference in the environment is a major driving force of evolution^1,2^. The ecology and behavior of many insect species are greatly affected by urbanization and the global increase in temperature^3^. This is supported by a number of studies that have shown evidence of a decrease in overall species richness and increase in the abundance of a few species that can thrive in urban environments^1,4–8^. While urbanization processes have a major impact on vector mosquito communities, some species such as *Aedes aegypti* and *Culex quinquefasciatus* have adapted to urban environments, which enabled them to thrive and benefit from these anthropogenic changes. Over the last decades, the distribution and abundance of these species have increased substantially^9–11^, leading to the emergence and re-emergence of many vector-borne diseases^12–15^. While some malaria vector species tend to seek hosts and breeding habitats in forested areas (e.g., *Anopheles triannulatus*, *An. oswaldoi*, and *An. cruzii*), others, such as *Anopheles darlingi*, are rarely found in forested areas but are frequently found in villages and can benefit from deforestation^16,17^.

The Brazilian Atlantic Forest is one of the most endangered ecosystems in the world^18^. This important biome is characterized by dense, humid forest with a high degree of biodiversity and endemism^19,20^. Historically, the Brazilian Atlantic Forest has undergone deforestation and fragmentation as a result of urbanization, industrialization, and agricultural expansion^21^. However, this process has intensified over the last decades, leading to increasing devastation and biodiversity loss in this biome^21,22^.

The Atlantic Forest is an important malaria transmission hotspot^23,24^ and is home to *Anopheles* (*Kerteszia*) *cruzii* Dyar and Knab, a neotropical mosquito found abundantly in this biome, where it is the main vector of simian and human malaria. This mosquito has a close relationship with plants from the *Bromeliaceae* family (bromeliads)^25^ as it uses the water accumulated in the bromeliad water tanks as its primary/exclusive breeding habitat^26^. For this reason, malaria in the Atlantic Forest has been known historically as “bromeliad malaria”^25,27^. Fragmentation of the Atlantic Forest ultimately reduces the number of natural malaria hosts (monkeys) and dead-end hosts in this region, increasing the contact between mosquito vectors and humans^23,28^. Some studies have found an association between anthropogenic changes in the environment and the occurrence of human malaria, asymptomatic plasmodial infections and zoonotic malaria transmission in the Atlantic Forest^28–31^.

Because of this species’ close relationship with the Atlantic Forest environment and because of its acrodendrophilic habits (i.e., its preference for inhabiting tree canopies), the ecology and phenotype of *An. cruzii* are believed to be affected by the fragmentation of the Atlantic Forest biome^30,32,33^. Interestingly, in environments where *An. cruzii* has been found in abundance only in tree canopies but not at ground level, cases of human malaria are rarely reported despite a high prevalence of malaria among nonhuman primates in the same areas^34^. However, in areas where the mosquito is found not only in the tree canopy but also at ground level, a higher prevalence of both human and simian malaria has been observed^34^. *Anopheles cruzii* blood feeds primarily on howler monkeys in tree canopies and on monkeys and humans at ground level. Hence, the species’ vertical dispersal increases the exposure of humans to *Plasmodium*^31,34–36^. The species was also found to be more active at ground level in areas with a greater edge effect and greater loss of forested areas^37^, a factor which may contribute to the zoonotic transmission of *Plasmodium*.

The presence of *An. cruzii* breeding sites in bromeliads in the urban environment has been reported previously^38,39^; however, this mosquito is considered a non-synanthropic species because although it can invade houses in search of blood meals, its natural breeding habitat is in forested areas^40,41^. Previous studies reported a high frequency of *An. cruzii* in peridomestic sites during an asymptomatic malaria outbreak in the Atlantic Forest and found that humans were a primary blood-meal source, indicating that this mosquito exhibits anthropophilic behavior^42^. The presence of *An. cruzii* in urban, peri-urban and sylvatic environments demonstrates the dispersive behavior of the species, as it can be found in different ecotypes, and its adaptation to anthropogenically modified areas close to forest fragments^33,37,39^.

Anthropogenic changes in Atlantic Forest ecosystems may have led to 185 cases of autochthonous human malaria between 2007 and 2017 in the city of São Paulo, the largest and most populous city in Brazil^43,44^. This hypothesis is supported by several autochthonous *P. vivax* malaria cases detected in the southern region of the city in the last decades^23,29^ and by previous studies in which *An. cruzii* was collected in abundance in tree canopies and at ground level in the region^37,45^. Furthermore, in a previous study carried out in the same region, *An. cruzii* was found naturally infected with *P. vivax* and *P. malariae*^29^.

Despite the epidemiological importance of this mosquito species in *Plasmodium* transmission and its high abundance in densely populated areas in the city of São Paulo, there is a dearth of studies assessing how anthropogenic changes in the environment affect the ecology and behavior of *An. cruzii* and how such changes may be driving patterns of malaria transmission. Moreover, to our knowledge no genetic studies have yet attempted to understand how *An. cruzii* is adapting locally to the increased anthropogenic modification and destruction of its natural habitats^24^. Single nucleotide polymorphisms (SNPs) are informative molecular markers used to analyze genetic differences between populations on micro- and macro-geographic and evolutionary scales^46,47^ and can be used to examine neutral genome regions and regions under selection, increasing the sensitivity and specificity of this type of analysis^48^. Various population studies in the literature have analyzed SNPs in *Anopheles* species from around the world^49–51^, and the structure of populations of *An. darlingi*, the main malaria vector in the Amazon region, has been analyzed on microgeographic and macrogeographic scales with the aid of SNPs in several Brazilian studies^52–55^. These studies show that this genetic tool is reliable and sensitive enough to reveal genetic differences using a fraction of the samples required for other molecular markers.

**Figure 1:**
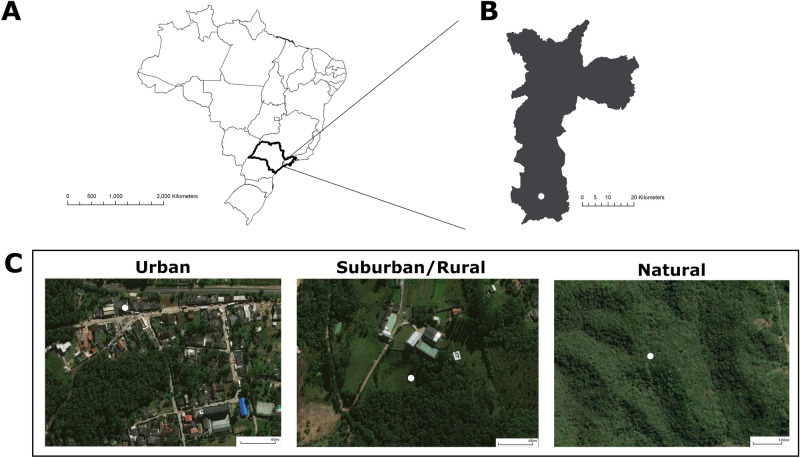
Map of the *Anopheles cruzii* sampling locations in the city of São Paulo, Brazil. (**a**) Map of Brazil showing the locality of the city of São Paulo in the state of São Paulo in the southeastern region of the country. (**b**) Map of the city of São Paulo showing the locality of the subdistrict of *Parelheiros* where the *An. cruzii* collections occurred. (**c**) Satellite images of the *An. cruzii* sampling locations. From left to right: Urban—the *Engenheiro Marsilac* neighborhood; Suburban/Rural—a transition area between Atlantic Forest remnants and a cattle range; and Natural—Atlantic Forest remnants on private property. The map of vegetation remnants of the Atlantic Forest Biome in the municipality of São Paulo is available at https://geosampa.prefeitura.sp.gov.br/PaginasPublicas/_SBC.aspx.

Understanding how anthropogenic changes in natural areas affect *An. cruzii* is essential for the development of more effective mosquito control strategies and, on a broader scale, for malaria-elimination efforts in Brazil. The objective of this study was therefore to genetically characterize populations of *An. cruzii* collected over 17 months in three different environments that have undergone different degrees of anthropogenic impacts and were classified as Natural, Suburban/Rural, and Urban (Fig. 1). We tested five hypotheses using a cross-sectional and longitudinal design to assess genetic structure in sympatric *An. cruzii* specimens from tree-canopy and ground-level habitats. Additionally, we assessed cross-sectional genetic structure and microevolution of *An. cruzii* specimens from environments with different degrees of anthropogenic modifications. Hypothesis-1—There is genetic structure between tree-canopy and ground-level *An. cruzii* populations; Hypothesis-2—There is genetic structure between tree-canopy and ground-level *An. cruzii* populations in each environment (Natural, Suburban/Rural and Urban); Hypothesis-3—There is cross-sectional genetic structure between the different environments (Natural, Suburban/Rural and Urban); Hypothesis-4—There is microevolutionary structure between populations over time; and Hypothesis-5—There is microevolutionary structure between populations in each environment (Natural, Suburban/Rural, and Urban).

## Results

**Figure 2:**
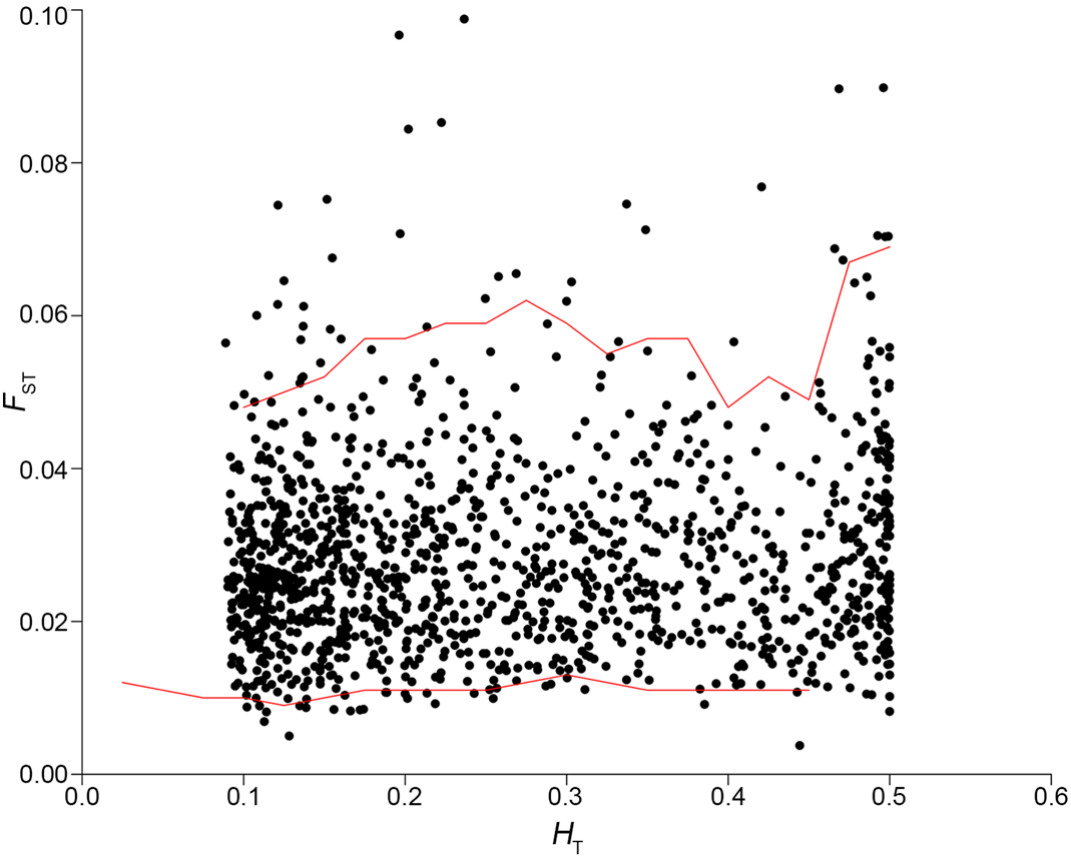
Detection of outlier SNPs. Graph of the distribution of heterozygosity values in relation to *F*_ST_ values of all filtered SNPs. Each locus is represented by a black dot in the graph. The red lines show 95% smoothed quantiles calculated by *fsthet*.

A total of 21,678 SNPs were genotyped in 380 specimens of *An. cruzii* (Table 1) by SNPsaurus (SNPsaurus, Oregon, USA). Filtering of this dataset (for quality, read depth, LD, MAF and indels) resulted in a set of markers with 1,313 SNPs. Outlier detection based on the *fsthet* method identified 78 (approx. 6%) loci that are possibly under positive selection^56^ (Fig. 2). These loci were removed from the subsequent analysis. The remaining 1235 SNPs were assumed to be neutral although their neutrality could not be directly proven.

**Table 1:** *Anopheles cruzii* sampling information.

Collection site	Trap	Number of specimens*		Geographic coordinates		Vegetation cover (%)
		2016	2017	Latitude	Longitude	
Natural	CDC canopy	30	25	23° 56.378′S	46° 41.659′W	92
	CDC ground	30	–			
	Shannon	30	26			
Suburban/rural	CDC canopy	22	25	23° 54.556′S	46° 42.167′W	71
	CDC ground	30	–			
	Shannon	30	30			
Urban	CDC canopy	18	9	23° 54.395′S	46° 42.486′W	63
	CDC ground	15	–			
	Shannon	30	30			

*Specimens randomly selected from collections conducted once a month from February 2016 to July 2017.

Comparisons of the test for Hardy–Weinberg equilibrium (HWE) for all the populations showed that expected heterozygosity was higher than observed heterozygosity (Ho) (Table 2). However, according to the *P* values, the deviations from HWE were not statistically significant.

**Table 2:** Genetic descriptors for 1235 SNPs in *Anopheles cruzii* populations for all tested hypotheses.

Hypothesis	Area	Population	Ho	He	HWE (non-corrected *P* value)	HWE (corrected *P* value)
Hypothesis 1	All areas	Canopy	0.192	0.2647	0.2592	0.3099
		Ground	0.1907	0.2638	0.2442	0.2888
Hypothesis 2	Natural	Canopy	0.1963	0.269	0.4423	0.5642
		Ground	0.1862	0.2643	0.4349	0.5367
	Suburban/rural	Canopy	0.187	0.2717	0.4487	0.5562
		Ground	0.1994	0.2638	0.4855	0.6297
	Urban	Canopy	0.2119	0.2709	0.618	0.7587
		Ground	0.2057	0.2872	0.5587	0.6597
Hypothesis 3	Natural		0.1892	0.2648	0.133	0.149
	Suburban/rural		0.1889	0.2634	0.1342	0.1499
	Urban		0.1897	0.2626	0.1927	0.2233
Hypothesis 4	All areas	2016	0.1842	0.2627	0.122	0.1357
		2017	0.1921	0.2645	0.0833	0.0898
Hypothesis 5	Natural	2016	0.1853	0.2639	0.1895	0.2172
		2017	0.1966	0.2658	0.3523	0.4389
	Suburban/rural	2016	0.1853	0.2622	0.2039	0.2354
		2017	0.1946	0.265	0.3206	0.3938
	Urban	2016	0.1853	0.2639	0.1894	0.2171
		2017	0.1966	0.2658	0.3523	0.4389

Observed heterozygosity (Ho), Expected heterozygosity (He), Hardy–Weinberg equilibrium (HWE).

## Hypothesis 1 and Hypothesis 2

### Genetic structure

Global statistics performed to measure population structure (*D*^57^, *F*_ST_ and *G*’’_ST_^58^) and the inbreeding coefficient (*F*_IS_) between populations, yielded low values that were not statistically significant for the tests of hypothesis 1, indicating that there is no genetic structure between pooled canopy and ground-level populations from the Natural, Suburban/Rural and Urban environments (Table 3). All population structure estimates for the tests of hypothesis 2 for populations from Natural and Suburban/Rural environments also yielded low values that were not statistically significant (Table 3). In contrast, estimates for the Urban population revealed low and statistically significant values for *D*, *F*_ST_ and *F*_IS_, except for the estimator *G”*_ST_, which showed moderate genetic structure, albeit with a non-significant *P* value (Table 3). Pairwise estimates of *F*_ST_, *G”*_ST_ and *D* among all populations in hypothesis 2 revealed significant estimates for the same pairs of populations, Natural ground x Suburban/Rural ground, Natural ground x Urban ground, and Urban canopy x Urban ground (S3 Table). Suggesting that the ground-level population in all areas is more structured than the tree-canopy populations. However, after the correction for multiple tests, all *P* values for all tests became non-significant. Analysis of molecular variance (AMOVA) carried out to detect population differentiation, showed that 99.83% and 99.24% of the variance for the tests of hypothesis 1 and 2, respectively, were estimated to be within populations, with non-significant *P* values (S4 Table). These results indicate that there is no population structure between the populations from the Natural, and Suburban/Rural environment. For the Urban environment, however, the results revealed that there may be genetic structure at a low hierarchical level.

**Table 3:** Population structure statistics. Global estimates of *D*, *F*_ST_, *F*_IS_ and *G”*_ST_ for all SNPs for the tests of Hypothesis 1 and Hypothesis 2.

Hypothesis	Area	Population structure			
		Statistics	Estimate	Non-corrected *P* value	Corrected *P* value
Hypothesis 1	All areas	*D*	0.000229923	0.6183816	0.848255
		*F*_*ST*_	− 0.000702856	0.8941059	0.895105
		*F*_*IS*_	− 0.000989916	0.8951049	0.895105
		*G''*_*ST*_	0.02421096	0.8941059	0.895105
Hypothesis 2	Natural	*D*	0.000526055	0.7582418	0.863137
		*F*_*ST*_	3.46145E − 05	0.4335664	0.846953
		*F*_*IS*_	4.7792E − 05	0.4335664	0.846953
		*G''*_*ST*_	0.06047334	0.4285714	0.846953
	Suburban/rural	*D*	0.000759986	0.3396603	0.846953
		*F*_*ST*_	0.000353953	0.3876124	0.846953
		*F*_*IS*_	0.000509479	0.3876124	0.846953
		*G''*_*ST*_	0.07533064	0.5314685	0.848255
	Urban	*D*	0.001501095	**0.008991009**	0.210646
		*F*_*ST*_	0.004968052	**0.040959041**	0.210646
		*F*_*IS*_	0.007001884	**0.040959041**	0.210646
		*G''*_*ST*_	0.1256977	0.065934066	0.296703

Statistically significant *P* values (> 0.05) are shown in bold. Hypothesis 1: Comparison of tree-canopy (70) and ground-level (75) *Anopheles cruzii* populations from all areas. Hypothesis 2: comparison of *Anopheles cruzii* populations from tree canopy and ground level separated by area classified according to the degree of anthropogenic modification (Natural: 30/30, suburban/rural: 22/30 and urban: 18/15).

### Multivariate statistics

**Figure 3:**
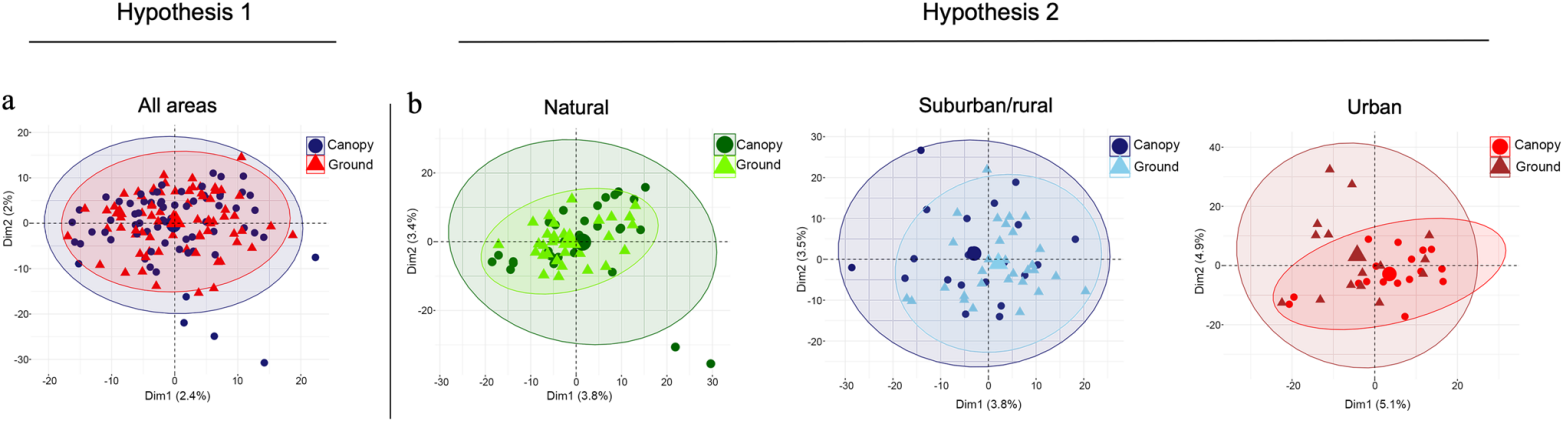
Genetic variation of tree-canopy and ground-level *Anopheles cruzii* populations using principal component analysis. (**a**) Hypothesis 1: comparison of tree-canopy (70) and ground-level (75) *Anopheles cruzii* populations from all areas. (**b**) Hypothesis 2: comparison of *Anopheles cruzii* populations from tree canopy and ground level separated by area classified according to the degree of anthropogenic modification (natural: 30/30, suburban/rural: 22/30 and urban: 18/15). Data were transformed using PCA and plotted as a function of the first two principal components. In parenthesis: number of specimens used in the analyses.

Principal component analysis (PCA) performed to assess genetic diversity among sampled populations revealed a high degree of overlapping in the test to verify hypothesis 1. The data variance explained by PC1 and PC2 of PCA was 2.4% and 2%, respectively (Fig. 3a). Overall, the population from the canopy habitat was more diverse and had more outliers. In the test of hypothesis 2, the data variances explained by PC1 and PC2 for the Natural population were 3.8% and 3.4%, respectively (Fig. 3b). There is a high degree of overlapping in the plot, and the mosquitoes collected at the canopy are genetically more diverse than the ones collected at ground-level. The data variances explained by PC1 and PC2 for the Suburban/Rural population were 3.8% and 3.5%, respectively (Fig. 3b). The plot shows overlapping, indicating similarity, and that both populations were highly dispersed. For the Urban population, the data variances explained by PC1 and PC2 were 5.1% and 4.9%, respectively (Fig. 3b). In the urban environment plot the ground-level population is more diverse than the canopy population, contrasting with the trend seen in the Natural and Suburban/Rural environments.

### Bayesian cluster analysis

**Figure 4:**
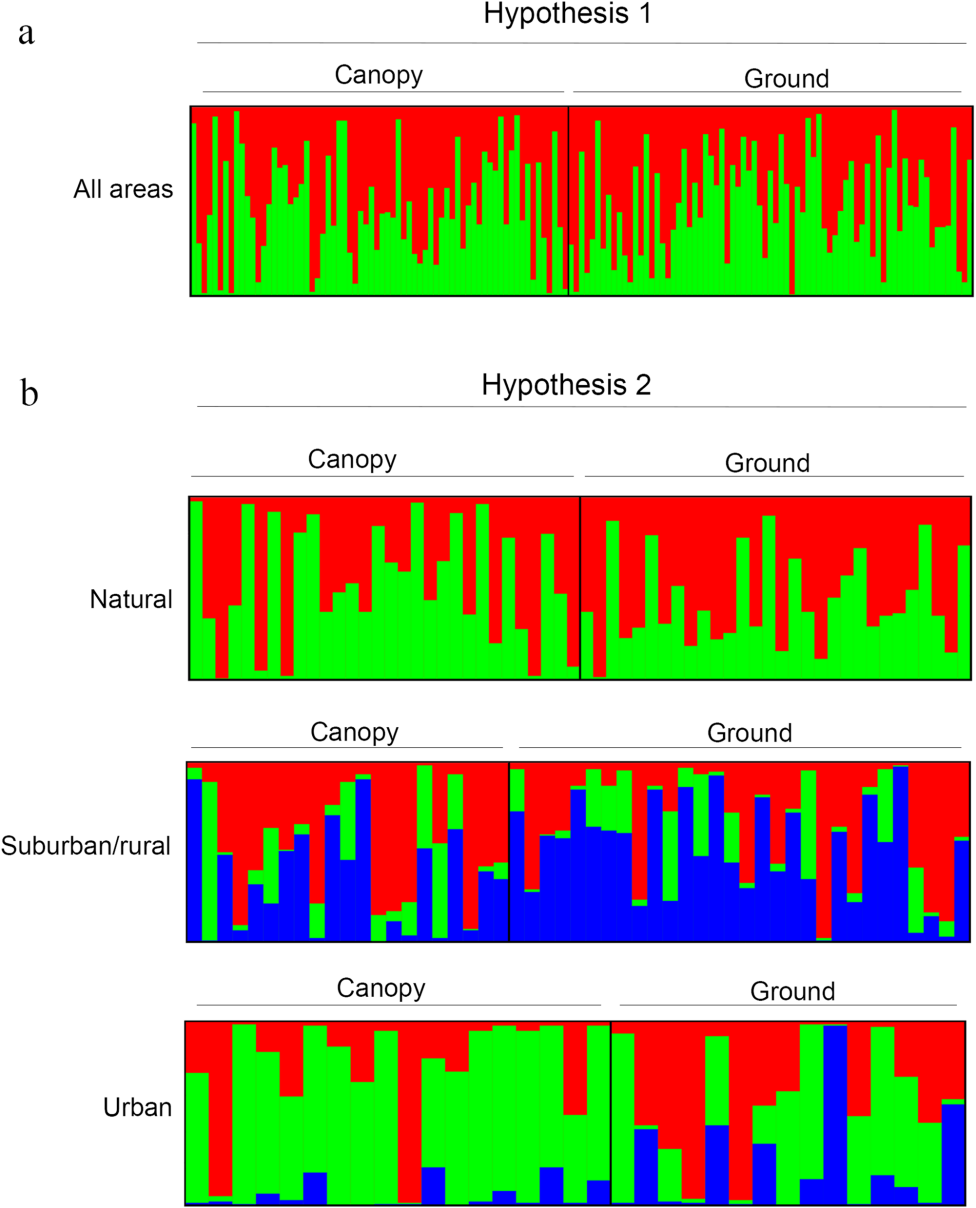
Genetic structure of tree-canopy and ground-level *Anopheles cruzii* populations in São Paulo. (**a**) Hypothesis 1: comparison of tree-canopy (70) and ground-level (75) *Anopheles cruzii* populations from all areas. (**b**) Hypothesis 2: comparison of tree-canopy and ground-level *Anopheles cruzii* populations separated by area classified according to the degree of anthropogenic modification (natural: 30/30, suburban/rural: 22/30 and urban: 18/15). Each of the individuals from the two populations collected in different areas is represented by a vertical line divided into different colored segments. The length of each segment represents the probability of the individual belonging to the genetic cluster represented by that color. In parenthesis: number of specimens used in the analyses.

The results of the Bayesian cluster analysis performed to infer population structure and subsequent application of the Evanno method, which identifies genetically homogeneous groups of individuals, revealed low structure in the *An. cruzii* populations studied (Fig. 4). For hypothesis 1, the most likely number of genetic groups was K = 2 according to the ΔK estimator. The STRUCTURE plot revealed two genetic groups across the two populations without a significant genetic structure between them (Fig. 4a). For hypothesis 2, the most likely number of groups for the Natural population was K = 2 according to the ΔK estimator. The STRUCTURE plot showed genetic groups across the two populations without a clear genetic structure between them.

The most likely number of groups for the Suburban/rural population was K = 3 according to the ΔK estimator. The STRUCTURE plot revealed three genetic groups well distributed between the two populations, and the canopy population showed slightly more variation in group membership than the ground-level population. The most likely number of groups for the Urban population was K = 3 according to the ΔK estimator. The STRUCTURE plot revealed three genetic groups which varied in membership between sympatric canopy and ground-level populations. While the canopy population is less structured with a predominance of the color green, the ground-level population appears to be more structured with an equal prevalence of the three genetic groups (Fig. 4b).

## Hypothesis 3, Hypothesis 4 and Hypothesis 5

The tests for hypotheses 3, 4, and 5 did not reveal significant genetic structuring between populations, indicating that the three hypotheses should be rejected. Although genetic estimators (*D*, *F*_ST_, and *G”*_ST_) and inbreeding coefficient (*F*_IS_) showed low estimates for the tests of hypothesis 3 with significant *P* value, after correction for multiple tests all *P* values became non-significant. Furthermore, the lack of structuring was corroborated by PCA and Bayesian analysis, which showed no signs of cross-sectional structuring between the populations from the Natural, Suburban/Rural and Urban environments (S1 Table; S1 Fig). Mantel test showed no correlation between genetic distance (*F*_ST_/(1 − *F*_ST_)) and geographic distance between Natural, Suburban/Rural and Urban environments, indicating no evidence of isolation by distance (IBD) between these populations (r =  − 0.1449955; *P* = 0.6689). The analyses indicated that the *An. cruzii* populations collected in 2016 and 2017 did not show temporal variation when the environments with different degrees of anthropogenic modifications were analyzed pooled and separately (S2 Table; S2 Fig). AMOVA showed that more than 99% of the variance was estimated to be within populations in the tests for all hypotheses with non-significant *P* values (S4 Table).

## Discussion

Deforestation and fragmentation of forests can have a negative impact on the abundance and survival of *An. cruzii*, a primary vector of human malaria in the Atlantic Forest, as this species is closely associated with forested areas^59^. However, because of its extremely anthropophilic nature, anthropogenic modifications can increase contact between this species and humans. We tested five hypotheses using a cross-sectional and longitudinal design to assess genetic structuring in sympatric *An. cruzii* populations and cross-sectional genetic structuring and microevolution in populations of this species from environments with different degrees of anthropogenic modifications.

Our findings suggest that *An. cruzii* has weaker fine-scale genetic structure than other *Anopheles* vectors in Brazil^53,55^. Nevertheless, the tests of hypothesis 2, in which *An. cruzii* populations collected in tree canopies and at ground level habitats in each environment were compared, yielded interesting results. PCA showed a different pattern of diversity in the Natural and Suburban/Rural environments, where the canopy habitat populations were more diverse, while in the Urban environment the ground-level habitat population appeared to be more diverse. To our knowledge, this is the first study in which *An. cruzii* populations collected in tree canopies and at ground level have been compared genetically. Our results show a clear structuring pattern between tree-canopy and ground-level populations from the Urban environment, and this was corroborated by all the analyses carried out. PCA and Bayesian cluster analyses showed that the urban ground-level population is more diverse and structured than the urban tree-canopy population.

While SNPs are reliable molecular markers for analyzing structure in mosquito populations on a microgeographic scale^53,60^, the SNP analyses showed higher expected heterozygosity than observed heterozygosity in all the hypotheses tested, indicating low levels of heterozygosis among the populations. Moreover, there was no evidence of cross-sectional structure between populations from the Natural, Suburban/Rural or Urban environments according to the PCA, Bayesian analysis and AMOVA, indicating that the differences between populations are very subtle, and only when closely related populations were compared were signs of local-level structuring observed. Although the genetic structure statistics (*D*, *F*_ST_ and *G”*_ST_) and the inbreeding coefficient (*F*_IS_) yielded statistically significant values for the test of hypothesis 3, all the estimated values were quite low indicating low genetic structure. Furthermore, the significant *F*_IS_ values could indicate that the heterozygosity in *An. cruzii* populations could be reduced by inbreeding of individuals. However, after correction for multiple tests, all *P* values became non-significant. It is important to consider that this study is investigating subtle variations in sympatric *An. cruzii* populations in both microgeographic and fine-temporal scales, which in turn can impact the significance of *P* values for multiple tests as it does not consider the power of the tests^61,62^.

The specimens analyzed here were collected in three different environments and were considered three different populations. However, as there are no geographic barriers, such as mountains, bodies of water, or a large canyon between the environments, our results indicated that the specimens represent a large, well-distributed population with a high overall genetic homogeneity. Previous studies carried out with mosquitoes from Brazil on a micro-geographic scale, suggest that native species exhibited weaker genetic structure while invasive species were shown to be more genetically structured and diverse^10,11,63,64^.

In the present study, *An. cruzii* populations showed no signs of genetic structure and variation when analyzed over time although microevolutionary changes, reflected as a variation in wing-shape over time in this species in the same areas, appear to vary greatly in urban environments compared with sylvatic and peri-urban environments^33^*.* Molecular and phenotypic markers are often contradictory, and in a previous study by our group geometric morphometrics showed clear structuring over time in *An. cruzii* but genetic analysis did not indicate significant structuring in the same populations^33^. Studies comparing the results of analyses using wing geometry and microsatellite markers in mosquitoes have shown that wing patterns change faster than genetic patterns^65^. While shape in mosquitoes is determined by multiple genes and their expression, SNPs can be filtered to ensure that they are independent, neutral and bi-allelic markers. Hence, differences in outcomes are to be expected for these two approaches^66,67^.

The loss of genetic diversity in native populations may be associated with anthropogenic modifications in the environment (e.g., deforestation, agriculture and urbanization)^1,53,68^. In addition, there is growing evidence that animal and plant populations experience divergent selection in urban and nonurban environments, which can explain the increased levels of genetic diversity in the population from ground-level habitat in the urban environment in the present study^1,5^. While there is no evidence of the presence of *An. cruzii* in highly urbanized metropolitan areas^69^, this species is constantly found in human dwellings close to forest fragments and in ornamental bromeliads in the anthropic environment^32,33,39,70^. Although *An. cruzii* is a bromeliad-specialist and its abundance is clearly associated with humidity, forest cover and bromeliad availability, studies have shown that anthropogenic modifications in natural areas can drive vertical dispersal in this mosquito and a variation in its phenotype and genotype, which in turn may drive the dynamics of *Plasmodium* transmission involving this important vector^33,37^.

A previous study showed that although anthropogenic changes in the environment can lead to a reduction in the abundance of *An. cruzii*, this species is found more frequently at ground level habitat in environments with increased deforestation due to human use^37^, indicating that the acrodendrophily of *An. cruzii* can be affected by human modifications in natural areas and areas close to dwellings^37,71^. These ecological findings corroborate the findings of our genetic study, suggesting that the presence of *An. cruzii* at ground level could be associated with proximity to anthropogenically modified environments. This varying acrodendrophilic behavior of *An. cruzii* is known to have important epidemiological implications for the dynamics of malaria transmission^28,34^.

Our findings suggest that the changes promoted by human activities play an essential role in the vertical dispersal of *An. cruzii*. Anthropogenic fragmentation of the Atlantic Forest biome leads to a reduction in natural hosts for this mosquito to blood feed on, which in tree canopies would be mainly birds and nonhuman primates^24,71,72^. This reduction may be driving *An. cruzii* to look for blood sources at ground level in places with human dwellings close to forest patches, which corroborates the anthropophilic behavior of *An. cruzii* and provides further evidence for the transmission of human and simian *Plasmodium* to humans living in the vicinity of forested areas^28,37,71^.

Furthermore, previous studies have shown that in some areas *An. cruzii* is more present inhabiting tree canopies, while in other areas it can be found in similar numbers in tree canopies and at ground level^37,73^. In a study conducted at the *Horto Florestal da Cantareira*, São Paulo, 99% of *An. cruzii* specimens collected were found in the tree canopies and despite the high malaria prevalence among the simian population there were no human cases of malaria^73^, whereas in the state of Santa Catarina, in the south of Brazil, a region where simian and human malaria have been reported, *An. cruzii* was found abundantly in both tree canopies and at ground level^73^. The same phenomenon is likely occurring in Parelheiros, a region with low malaria endemicity in the city of São Paulo where *An. cruzii* was collected in high numbers in both tree canopies and at ground level habitats^29,37,45^.

The different patterns of *An. cruzii* acrodendrophily led previous authors to believe that this species could represent two different species^34^. After collecting *An. cruzii* specimens from tree canopies and ground level, Deane et al. (1984) marked them with dye and released them to test their pattern of vertical dispersal. When they had collected the marked specimens again, the authors noticed that mosquitoes released at ground level were collected in tree canopies and vice-versa. They suggested that the mosquitoes in the study area belonged to the same species but did not dismiss the possibility of *An. cruzii* being two species^34^. Although there is probably still movement between *An. cruzii* from tree canopies to ground level and vice-versa, our results suggest that in areas close to human dwellings, *An. cruzii* populations are more diverse and genetic structured at ground level.

Contrarily to this study, previous studies conducted in different regions of Brazil found significant genetic variation between populations of *An. cruzii*^74–76^. Evidence of structuring in *An. cruzii* populations from high altitudes and low altitudes were observed using both nuclear and mitochondrial markers^74,75^. Furthermore, it has been suggested that *An. cruzii* may be a complex with at least two sibling species occurring in the southern and southeastern regions of Brazil^76,77^. However, these studies were conducted using molecular markers other than SNPs and on a larger scale than the present study. The present study is not without limitations. The sampling was conducted in only one site of each type of habitat (Natural, Suburban/Rural and Urban) with no replications, therefore, the genetic differences found in this study, even though unlikely, could be due to a factor unique to that one site.

Effective vector-control strategies are very important to eradicate malaria. Because of their complex biology and ecology and similar genetic characteristics, *An. cruzii* populations are particularly difficult to control. The species moves from natural areas to domestic and peridomestic areas in search of blood sources, posing a challenge for control strategies based on integrated vector management^28,78^. In the past, deforestation and bromeliad destruction were common approaches adopted to reduce the population of this vector mosquito during malaria outbreaks, but these strategies are no longer acceptable^79^. Deforestation leads to loss of biodiversity, resulting in a reduction in the number of dead-end *Plasmodium* hosts in the Atlantic Forest and an increase in the likelihood of *Plasmodium* transmission to humans^23,28^.

Considering that *An. cruzii* is not a synanthropic species, a significant genetic structure between populations from Natural, Suburban/Rural, and Urban areas as well as tree-canopy and ground-level at the microgeographic scale indicates the disruption of its natural environments. Such presence of genetic variation in sympatric *An. cruzii* populations can be used as a proxy for ecosystem health, and to infer both the impact of deforestation and defaunation in its distribution as well as the extent of human exposure to this mosquito vector species. Environmental planning, forest conservation, and control of illegal human settlements and ecotourism in protected areas could play an important role in reducing the presence of this vector at ground level, resulting in turn in a likely reduction in plasmodial infections in the Atlantic Forest^23,28^.

## Conclusions

Our findings point to the existence of structuring between populations of *An. cruzii* from tree canopies and ground level habitats in the urban environment, with the ground-level population showing higher genetic variation. Although this study has some limitations, our results point to the possibility that anthropogenic modifications leading to habitat fragmentation may be driving the biology of *An. cruzii* and maintaining genetic diversity and structure in populations more present at ground level. Our study design could be used to assess the genetics of *An. cruzii* populations in malaria-transmission areas where this species is abundant at ground level.

## Material and methods

### Study area

The chosen study area was the Capivari-Monos conservation area in the subdistrict of Parelheiros, São Paulo, Brazil. Three environments with different levels of anthropogenic environmental modifications were chosen: Natural—Atlantic Forest remnants on private property; Suburban/Rural—a transition area between Atlantic Forest remnants and a cattle range; and Urban—the *Engenheiro Marsilac* neighborhood. The map of sampling locations was produced using ArcGIS 10.2 (Esri, Redlands, CA).

Vegetation cover at each collection site was identified using the "Map of vegetation remnants of the Atlantic Forest Biome in the municipality of São Paulo" available at https://geosampa.prefeitura.sp.gov.br/PaginasPublicas/_SBC.aspx. A one-kilometer buffer, defined around each georeferenced collection site, was used to characterize vegetation cover at each collection site. The area within each buffer was then used to measure the proportion of vegetation cover around each collection site. Measurements were made in QGIS 2.18 (https://www.qgis.org)^37^.

### Mosquito collections and identification of specimens

Mosquito collections were conducted once a month from February 2016 to July 2017. Adult specimens were collected using CO_2_-baited CDC light traps (CDC-LT)^80^ placed in tree canopies (at heights > 12 m) and at ground level (at a height of 1 m) for 14 h including dusk and dawn, when *An. cruzii* blood-feeding activity peaks. A Shannon light trap was also set up at each collection site for 2 h after dusk. Mosquitoes were collected over 17 months with the traps set in different locations within each collection site. When possible, 30 specimens collected throughout 17 months were randomly selected from each trap in each collection site to avoid bias introduced by analyzing siblings^81,82^. Mosquitoes were identified with the aid of taxonomic keys^26^ and stored in 1.5 mL tubes with isopropyl alcohol at room temperature until DNA was extracted.

### SNP genotyping

Genomic DNA was extracted from mosquitoes’ whole body using the DNeasy Blood and Tissue kit (Qiagen) following the manufacturer’s protocol. A Qubit Fluorometer (ThermoFisher, Dietikon, Switzerland) was used to determine DNA concentration, and electrophoresis on 1% agarose gel was performed to assess the quality and purity of the samples. Raw genomic DNA was then sent to SNPsaurus (SNPsaurus, Oregon, USA) to be converted into nextRAD genotyping-by-sequencing libraries^83^.

Using Nextera technology (Illumina, Inc), genomic DNA was simultaneously fragmented and tagged with short adapter sequences to the cleaved ends. The Nextera reaction was scaled to fragment 10 ng of genomic DNA, of which up to 5 ng was used for input to help dilute inhibitors. Fragmented DNA was then amplified using Phusion Hot Start Flex DNA Polymerase (New England Biolabs, Inc., Ipswich, MA) with one of the primers matching the adapter and extending 8 nucleotides into the genomic DNA with the selective sequence TGCAGGAG. Thus, only fragments starting with a sequence that can be hybridized by the selective sequence of the primer will be efficiently amplified. PCR conditions were as follows: 3 min at 72 °C, 3 min at 98 °C, followed by 25 cycles of 45 s at 98 °C and 1 min at 75 °C. The nextRAD libraries were sequenced on a HiSeq 4000 with four lanes of single-end 150 bp reads (University of Oregon).

The genotyping analysis were processed using SNPsaurus nextRAD custom scripts (SNPsaurus, Oregon, USA)^83,84^ that trimmed the reads with BBDuk (BBMap tools, https://sourceforge.net/projects/bbmap/). Next, a de novo reference genome was created by collecting 10 million reads equally divided between the samples and excluding reads using the thresholds of 7 and 700, which have been empirically determined to produce de novo reference loci that are useful for alignment^83^. The remaining loci were then aligned to each other to identify allelic loci and collapse allelic haplotypes into a single representative. All reads were mapped to the reference with an alignment identity threshold of 0.95 using BBMap (BBMap tools). Genotype calling was performed using SAMtools^85^ and BCFtools^86^. The variant call format (VCF) file was filtered to remove alleles with a population frequency of less than 3% and loci that were heterozygous in all samples or had more than 2 alleles in a sample, suggesting collapsed paralogs. The absence of artifacts was checked by counting SNPs at every nucleotide position read and verifying that the number of SNPs did not increase with reduced base quality toward the end of the read^87^.

### Statistical and genetic analysis

VCFtools (https://github.com/vcftools)^88^ was used to perform the final SNP filtering of the genotype data. The criteria established for locus filtering were based on published data^83,87^: loci were filtered to assess minimum base quality (30), read depth (20), linkage disequilibrium to select a single independent SNP per locus, minimum allele frequency of 5% across all populations, exclusion of loci with more than 5% of missing data, and removal of indels.

The filtered dataset was entered in PGDSpider to convert the data into STRUCTURE 2.3.4^89^ and Arlequin 3.5^90^ formats. Filtered loci were analyzed to produce smoothed quantiles that enabled the relationship between *F*_ST_ and heterozygosity in each locus to be determined. This analysis uses the raw empirical data^56^ to identify outlier loci which can be under selection. The analysis was carried out with the R package *fsthet*^56,91^.

After filtering and identification of outlier loci, the resulting independent and neutral SNPs were used to characterize the populations genetically. *Anopheles cruzii* specimens collected in three different environments with different levels of anthropogenic changes (Natural, Suburban/Rural and Urban) were considered three different populations and its respective SNPs were then separated according to each of the five hypotheses tested. In the tests of each hypothesis, each sample being compared was considered a different ‘population’. Data corresponding to each hypothesis were analyzed with the statistical and genetic analysis software. All analyses comparing tree-canopy and ground-level samples were carried out with specimens collected only in the year of 2016.

Observed heterozygosity (Ho), expected heterozygosity (He) and Hardy–Weinberg equilibrium (HWE) tests for all SNPs per population per hypotheses were performed in Arlequin v3.5^90^ to investigate heterozygosis among the populations. To assess the genetic variance between populations, we used the genetic structure estimators *D*^57^, *F*_ST_ and *G*’’_ST_^58^, and the inbreeding coefficient *F*_IS_ were estimated globally in R using the *StrataG* package^92^. Pairwise estimates of *D*, *F*_ST_ and *G*’’_ST_ among all populations in hypothesis 2 were conducted in R using the *StrataG* package^92^ to further investigate genetic structure between tree-canopy and ground-level populations. *P* values for all multiple tests were adjusted by applying the false discovery rate correction^93^ using the function *p.adjust* in R^91^. To assess genetic diversity among sampled population, we used the multivariate principal component analysis (PCA) carried out with *adegenet*^94^ and *ade4*^95^ in R. Analysis of molecular variance (AMOVA) was performed in Arlequin 3.5^90^ to detect population differentiation in all hypotheses. To test for isolation by distance for the populations in hypothesis 3, a Mantel test between genetic distance (*F*_ST_/(1 − *F*_ST_)) and geographic distance in kilometers was calculated with *ade4*^95^ package in R^91^ using 9,999 permutations.

Bayesian cluster analysis in STRUCTURE was performed with the *StrataG* package in R to infer population structure between populations in all tested hypotheses. The analysis was carried out assuming the Admixture model and correlated allelic frequencies among populations^52–54^. Run length was set to 100,000 MCMC replicates after a burn-in period of 100,000. The number of clusters (K) varied from 1 to 10, with 10 replicates for each value of K^83^. The most likely number of clusters was determined by the ΔK method^96^, which identifies genetically homogeneous groups of individuals, used in Structure Harvester^97^.

## Supplementary information

Supplementary Legends

Supplementary Information 2

Supplementary Information 3

Supplementary Information 4

Supplementary Information 5

Supplementary Information 6

Supplementary Information 7

## Data Availability

Data supporting the conclusions are included within the article. The dataset analyzed during the current study are available in the Mendeley Data repository, https://doi.org/10.17632/jd72b5v8vv.1.
