# Supplementary material for: The influence of anthropogenic habitat fragmentation on the genetic structure and diversity of the malaria vector Anopheles cruzii (Diptera: Culicidae)
Source: Sci Rep. 2020 Oct 22;10:18018. doi: 10.1038/s41598-020-74152-3 (PMC7581522; doi:10.1038/s41598-020-74152-3)
Supplement: Supplementary file 1 — Supplementary Legends [file 41598_2020_74152_MOESM1_ESM.docx]

**Supplementary Information**

**Tables**

**S1 Table. Population structure statistics.** Global estimates of *D*, *F*_ST_, *F*_IS_, and *G”*_ST_ for all SNPs for the tests of Hypothesis 3. **Hypothesis 3:** Cross-sectional comparison of *An. cruzii* populations from Natural (141), Suburban/Urban (137) and Urban (102) areas.

**S2 Table. Population structure statistics.** Global estimates of *D*, *F*_ST_, *F*_IS_, and *G”*_ST_ for all SNPs for the tests of Hypotheses 4 and 5. **Hypothesis 4:** Comparison of *An. cruzii* populations collected in 2016 (235) and 2017 (145) from all areas. **Hypothesis 5:** Comparison of *An. cruzii* populations collected in 2016 and 2017 separated by area classified according to the degree of anthropogenic modification (Natural: 90/51, Suburban/Rural: 82/55 and Urban: 63/39).

**S3 Table.** Pairwise genetic structure estimates (*F*_ST_, *G”*_ST_ and *D*) for all *Anopheles cruzii* populations in the tests of hypothesis 2. *P*-values non-corrected (below diagonal) and corrected (above diagonal) using the false discovery rate correction. **Hypothesis 2**: Comparison of *Anopheles cruzii* populations from tree canopy and ground level separated by area classified according to the degree of anthropogenic modification (Natural: 30/30, Suburban/Rural: 22/30 and Urban: 18/15).

**S4 Table.** Global AMOVA results based on 1,235 SNPs in the *Anopheles cruzii* populations for all tested hypotheses.

**Figures**

**S1 Fig. Genetic structure and variation of *Anopheles cruzii* populations from Natural, Suburban/Rural and Urban areas (Hypothesis 3).** Hypothesis 3: Cross-sectional comparison of *An. cruzii* populations from Natural (141), Suburban/Urban (137) and Urban (102) areas. A) Principal Component Analysis. B) Multi-locus Bayesian analysis implemented in STRUCTURE. In parenthesis: number of specimens used in the analyses.

**S2 Fig. Genetic structure and variation of *Anopheles cruzii* populations collected in 2016 and 2017 from Natural, Suburban/Rural and Urban areas (Hypotheses 4 and 5).** Hypothesis 4: Comparison of *An. cruzii* populations collected in 2016 (235) and 2017 (145) from all areas. Hypothesis 5: Comparison of *An. cruzii* populations collected in 2016 and 2017 separated by area classified according to the degree of anthropogenic modification (Natural: 90/51, Suburban/Rural: 82/55 and Urban: 63/39). A) Principal Component Analysis. B) Multi-locus Bayesian analysis implemented in STRUCTURE. In parenthesis: number of specimens used in the analyses.
