# Supplementary material for: The influence of anthropogenic habitat fragmentation on the genetic structure and diversity of the malaria vector Anopheles cruzii (Diptera: Culicidae)
Source: Sci Rep. 2020 Oct 22;10:18018. doi: 10.1038/s41598-020-74152-3 (PMC7581522; doi:10.1038/s41598-020-74152-3)
Supplement: Supplementary file 2 — Supplementary Information 2 [file 41598_2020_74152_MOESM2_ESM.docx]

**S1 Table. Population structure statistics.** Global estimates of *D*, *F*_ST_, *F*_IS_, and *G”*_ST_ for all SNPs for the tests of Hypothesis 3.

| **Hypothesis** | **Population structure** | | | |
| --- | --- | --- | --- | --- |
|  | **Statistics** | **Estimate** | **Non-corrected *P*-value** | **Corrected P-value** |
| Hypothesis 3 | *D* | 0.000199997 | **0.02397602** | 0.210646 |
|  | *F*_ST_ | 0.000507403 | **0.03496503** | 0.210646 |
|  | *F*_IS_ | 0.000722779 | **0.03496503** | 0.210646 |
|  | *G*''_ST_ | 0.01363214 | **0.03596404** | 0.210646 |

**Hypothesis 3:** Cross-sectional comparison of *An. cruzii* populations from Natural (141), Suburban/Urban (137) and Urban (102) areas.
