# Supplementary figures and images for: The influence of anthropogenic habitat fragmentation on the genetic structure and diversity of the malaria vector Anopheles cruzii (Diptera: Culicidae)
Source: Sci Rep. 2020 Oct 22;10:18018. doi: 10.1038/s41598-020-74152-3 (PMC7581522; doi:10.1038/s41598-020-74152-3)

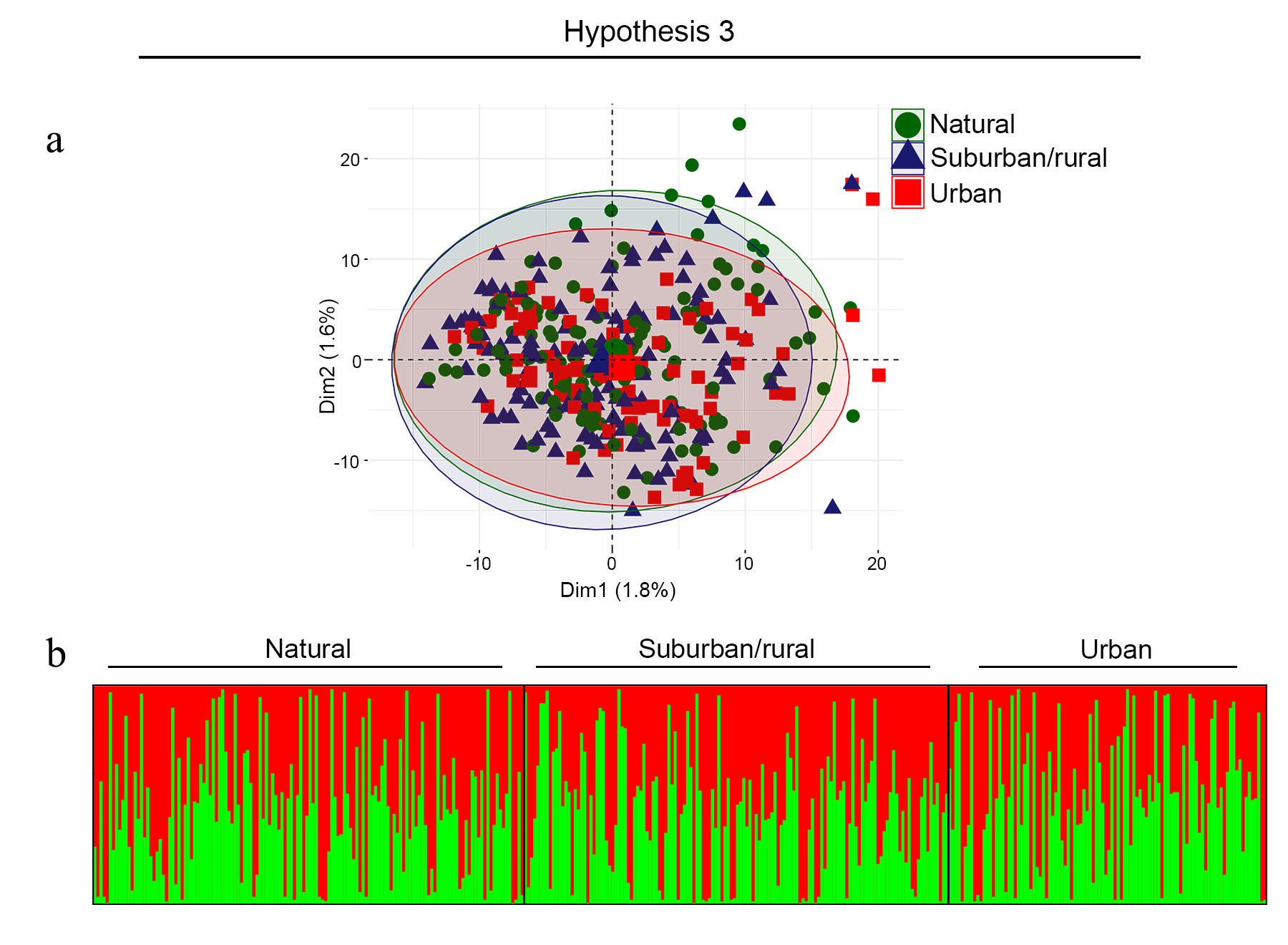

Supplement: Supplementary file 3 — Supplementary Information 3 [file 41598_2020_74152_MOESM3_ESM.tif]

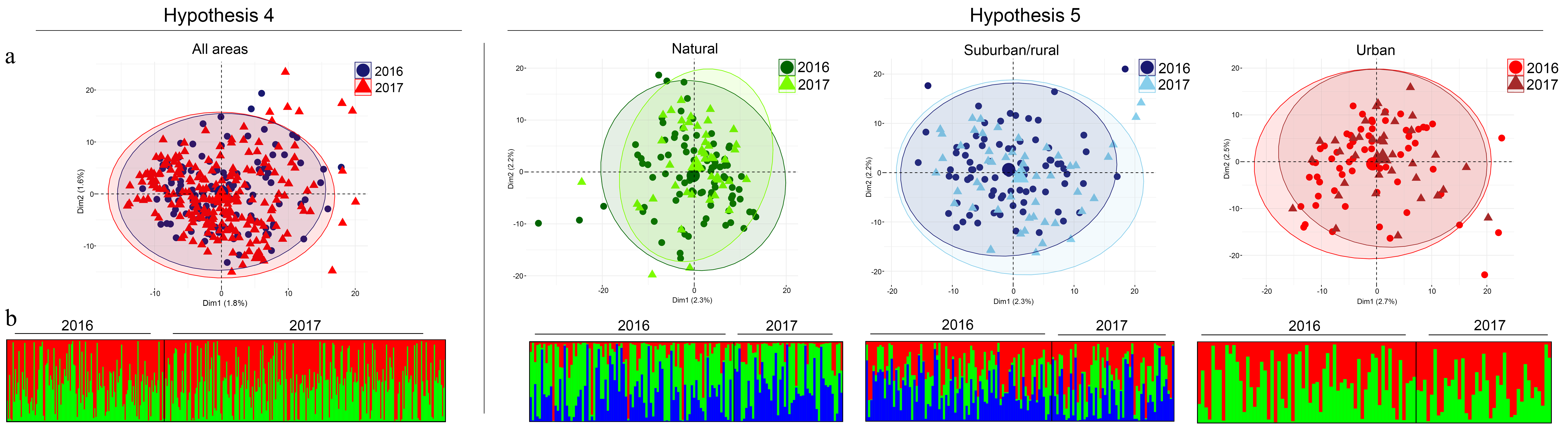

Supplement: Supplementary file 5 — Supplementary Information 5 [file 41598_2020_74152_MOESM5_ESM.tif]
