# Supplementary material for: The influence of anthropogenic habitat fragmentation on the genetic structure and diversity of the malaria vector Anopheles cruzii (Diptera: Culicidae)
Source: Sci Rep. 2020 Oct 22;10:18018. doi: 10.1038/s41598-020-74152-3 (PMC7581522; doi:10.1038/s41598-020-74152-3)
Supplement: Supplementary file 4 — Supplementary Information 4 [file 41598_2020_74152_MOESM4_ESM.docx]

**S2 Table. Population structure statistics.** Global estimates of *D*, *F*_ST_, *F*_IS_, and *G”*_ST_ for all SNPs for the tests of Hypotheses 4 and 5.

| **Hypothesis** | **Area** | **Population structure** | | | |
| --- | --- | --- | --- | --- | --- |
|  |  | **Statistics** | **Estimate** | **Non-corrected *P*-value** | **Corrected *P*-value** |
| Hypothesis 4 | All areas | D | 0.00010138 | 0.4705295 | 0.846953 |
|  |  | *F*_ST_ | 0.00021021 | 0.1898102 | 0.621197 |
|  |  | *F*_IS_ | 0.00029592 | 0.1898102 | 0.621197 |
|  |  | *G*''_ST_ | 0.01077554 | 0.1798202 | 0.621197 |
| Hypothesis 5 | Natural | *D* | 0.00028958 | 0.4185814 | 0.846953 |
|  |  | *F*_ST_ | -0.0003226 | 0.6423576 | 0.848255 |
|  |  | *F*_IS_ | -0.0004413 | 0.6413586 | 0.848255 |
|  |  | *G*''_ST_ | 0.0280301 | 0.7382617 | 0.863137 |
|  | Suburban/Rural | *D* | 0.00025936 | 0.5664336 | 0.848255 |
|  |  | *F*_ST_ | -0.0003234 | 0.6513487 | 0.848255 |
|  |  | *F*_IS_ | -0.0004483 | 0.6493506 | 0.848255 |
|  |  | *G*''_ST_ | 0.02742571 | 0.6783217 | 0.848255 |
|  | Urban | *D* | 0.00037651 | 0.4585415 | 0.846953 |
|  |  | *F*_ST_ | -0.000781 | 0.7882118 | 0.863137 |
|  |  | *F*_IS_ | -0.0011262 | 0.7912088 | 0.863137 |
|  |  | *G*''_ST_ | 0.03861435 | 0.6833167 | 0.848255 |

**Hypothesis 4:** Comparison of *An. cruzii* populations collected in 2016 (235) and 2017 (145) from all areas. **Hypothesis 5:** Comparison of *An. cruzii* populations collected in 2016 and 2017 separated by area classified according to the degree of anthropogenic modification (Natural: 90/51, Suburban/Rural: 82/55 and Urban: 63/39).
