# Supplementary material for: The influence of anthropogenic habitat fragmentation on the genetic structure and diversity of the malaria vector Anopheles cruzii (Diptera: Culicidae)
Source: Sci Rep. 2020 Oct 22;10:18018. doi: 10.1038/s41598-020-74152-3 (PMC7581522; doi:10.1038/s41598-020-74152-3)
Supplement: Supplementary file 6 — Supplementary Information 6 [file 41598_2020_74152_MOESM6_ESM.docx]

**S3 Table.** Pairwise genetic structure estimates (*F*_ST_, *G”*_ST_ and *D*) for all *Anopheles cruzii* populations in the tests of hypothesis 2. *P*-values non-corrected (below diagonal) and corrected (above diagonal) using the false discovery rate correction.

| *F_ST_* |  |  |  |  |  |  |
| --- | --- | --- | --- | --- | --- | --- |
|  | Natural Canopy | Natural Ground | Suburban/Rural Canopy | Suburban/Rural Ground | Urban Canopy | Urban Ground |
| Natural Canopy | - |  |  |  |  |  |
| Natural Ground | 3.99664E-05 | - |  |  |  |  |
| Suburban/Rural Canopy | 0.000378996 | -0.001383863 | - |  |  |  |
| Suburban/Rural Ground | 0.000123794 | 0.002974173 | -0.000121659 | - |  |  |
| Urban Canopy | 0.001233116 | 0.00077283 | 0.000517014 | 0.002154624 | - |  |
| Urban Ground | 0.001215787 | 0.003931104 | -0.001301464 | 0.001134612 | 0.004968052 | - |
|  |  |  |  |  |  |  |
| *P*-value |  |  |  |  |  |  |
|  | Natural Canopy | Natural Ground | Suburban/Rural Canopy | Suburban/Rural Ground | Urban Canopy | Urban Ground |
| Natural Canopy | - | 0.5475294 | 0.5475294 | 0.5475294 | 0.5475294 | 0.5475294 |
| Natural Ground | 0.45354645 | - | 0.7792208 | 0.2497503 | 0.5475294 | 0.2497503 |
| Suburban/Rural Canopy | 0.4025974 | 0.77922078 | - | 0.5475294 | 0.5475294 | 0.7117882 |
| Suburban/Rural Ground | 0.41758242 | **0.02197802** | 0.47452547 | - | 0.531968 | 0.5475294 |
| Urban Canopy | 0.25574426 | 0.33066933 | 0.37762238 | 0.14185814 | - | 0.2497503 |
| Urban Ground | 0.25874126 | **0.04995005** | 0.66433566 | 0.29470529 | **0.04195804** | - |
|  |  |  |  |  |  |  |
| *G"_ST_* |  |  |  |  |  |  |
|  | Natural Canopy | Natural Ground | Suburban/Rural Canopy | Suburban/Rural Ground | Urban Canopy | Urban Ground |
| Natural Canopy | - |  |  |  |  |  |
| Natural Ground | 0.0614446 | - |  |  |  |  |
| Suburban/Rural Canopy | 0.07232779 | 0.0696773 | - |  |  |  |
| Suburban/Rural Ground | 0.06272079 | 0.06595553 | 0.07253205 | - |  |  |
| Urban Canopy | 0.08822289 | 0.08716021 | 0.09767119 | 0.08984285 | - |  |
| Urban Ground | 0.09515144 | 0.09799507 | 0.10232992 | 0.09552987 | 0.12569767 | - |
|  |  |  |  |  |  |  |
| *P*-value |  |  |  |  |  |  |
|  | Natural Canopy | Natural Ground | Suburban/Rural Canopy | Suburban/Rural Ground | Urban Canopy | Urban Ground |
| Natural Canopy | - | 0.5681818 | 0.5681818 | 0.5681818 | 0.3716284 | 0.4538319 |
| Natural Ground | 0.45054945 | - | 0.8111888 | 0.3146853 | 0.3796204 | 0.3146853 |
| Suburban/Rural Canopy | 0.42057942 | 0.81118881 | - | 0.6518482 | 0.4813936 | 0.6518482 |
| Suburban/Rural Ground | 0.41658342 | **0.02097902** | 0.60839161 | - | 0.3716284 | 0.5681818 |
| Urban Canopy | 0.12387612 | 0.15184815 | 0.25674326 | 0.12087912 | - | 0.3696303 |
| Urban Ground | 0.21178821 | **0.04195804** | 0.58441558 | 0.45454545 | 0.07392607 | - |
|  |  |  |  |  |  |  |
| *D* |  |  |  |  |  |  |
|  | Natural Canopy | Natural Ground | Suburban/Rural Canopy | Suburban/Rural Ground | Urban Canopy | Urban Ground |
| Natural Canopy | - |  |  |  |  |  |
| Natural Ground | 0.000525359 | - |  |  |  |  |
| Suburban/Rural Canopy | 0.000680958 | 0.000708191 | - |  |  |  |
| Suburban/Rural Ground | 0.00056841 | 0.000805637 | 0.000747989 | - |  |  |
| Urban Canopy | 0.000781729 | 0.000791537 | 0.000990252 | 0.000925673 | - |  |
| Urban Ground | 0.001118214 | 0.001245518 | 0.001124403 | 0.001036674 | 0.001501095 | - |
|  |  |  |  |  |  |  |
| *P*-value |  |  |  |  |  |  |
|  | Natural Canopy | Natural Ground | Suburban/Rural Canopy | Suburban/Rural Ground | Urban Canopy | Urban Ground |
| Natural Canopy | - | 0.8001998 | 0.7149993 | 0.688158 | 0.6743257 | 0.4620379 |
| Natural Ground | 0.8001998 | - | 0.6743257 | 0.1048951 | 0.6743257 | 0.1048951 |
| Suburban/Rural Canopy | 0.66733267 | 0.53946054 | - | 0.6543457 | 0.5972599 | 0.6543457 |
| Suburban/Rural Ground | 0.5964036 | **0.01598402** | 0.35164835 | - | 0.3596404 | 0.4620379 |
| Urban Canopy | 0.5034965 | 0.45354645 | 0.27872128 | 0.0959041 | - | 0.1048951 |
| Urban Ground | 0.15484515 | **0.02097902** | 0.39260739 | 0.18481518 | **0.01298701** | - |

Hypothesis 2: Comparison of *Anopheles cruzii* populations from tree canopy and ground level separated by area classified according to the degree of anthropogenic modification (Natural: 30/30, Suburban/Rural: 22/30 and Urban: 18/15).
