# Supplementary material for: The influence of anthropogenic habitat fragmentation on the genetic structure and diversity of the malaria vector Anopheles cruzii (Diptera: Culicidae)
Source: Sci Rep. 2020 Oct 22;10:18018. doi: 10.1038/s41598-020-74152-3 (PMC7581522; doi:10.1038/s41598-020-74152-3)
Supplement: Supplementary file 7 — Supplementary Information 7 [file 41598_2020_74152_MOESM7_ESM.docx]

**S4 Table.** Global AMOVA results based on 1,235 SNPs in the *Anopheles cruzii* populations for all tested hypotheses.

| **Hypothesis** | **Area** | **Source of variation** | **Degrees of freedom** | **Sum of squares** | **Variance components** | **Percentage variation** | ***P*-value** |
| --- | --- | --- | --- | --- | --- | --- | --- |
| **Hypothesis 1** | All areas | Among populations | 1 | 86.572 | 0.12080 Va | 0.17 | 0.49634 |
|  |  | Within populations | 288 | 19894.19 | 69.07705 Vb | 99.83 |  |
| **Hypothesis 2** | Natural | Among populations | 1 | 97.642 | 0.51250 Va | 0.76 | 0.07427 |
|  |  | Within populations | 118 | 7893.25 | 66.89195 Vb | 99.24 |  |
|  | Suburban/Rural | Among populations | 1 | 76.905 | 0.28397 Va | 0.45 | 0.58289 |
|  |  | Within populations | 102 | 6373.806 | 62.48829 Vb | 99.55 |  |
|  | Urban | Among populations | 1 | 33.787 | 0.23736 Va | 0.9 | 0.31402 |
|  |  | Within populations | 64 | 1665.228 | 26.01918 Vb | 99.1 |  |
| **Hypothesis 3** | Natural | Among populations | 2 | 193.941 | 0.09867 Va | 0.14 | 0.17994 |
|  | Suburban/Rural | Within populations | 749 | 54266.733 | 72.45225 Vb | 99.86 |  |
|  | Urban |  |  |  |  |  |  |
| **Hypothesis 4** | All areas | Among populations | 1 | 98.81 | 0.07607 Va | 0.1 | 0.19152 |
|  |  | Within populations | 750 | 54361.864 | 72.48249 Vb | 99.9 |  |
| **Hypothesis 5** | Natural | Among populations | 1 | 90.757 | 0.15338 Va | 0.21 | 0.47901 |
|  |  | Within populations | 274 | 19605.196 | 71.55181 Vb | 99.79 |  |
|  | Suburban/Rural | Among populations | 1 | 84.431 | 0.10694 Va | 0.15 | 0.71737 |
|  |  | Within populations | 270 | 19035.859 | 70.50318 Vb | 99.85 |  |
|  | Urban | Among populations | 1 | 90.757 | 0.15338 Va | 0.21 | 0.48138 |
|  |  | Within populations | 274 | 19605.196 | 71.55181 Vb | 99.79 |  |
